# Correlation between p38 mitogen-activated protein kinase and human telomerase reverse transcriptase in sarcomas

**DOI:** 10.1186/1756-9966-31-5

**Published:** 2012-01-16

**Authors:** Toshihiro Matsuo, Shoji Shimose, Tadahiko Kubo, Jun Fujimori, Yuji Yasunaga, Takashi Sugita, Mitsuo Ochi

**Affiliations:** 1 Department of Orthopaedic Surgery, National Hospital Organization Kure Medical Center and Chugoku Cancer Center: 3-1, Aoyamacho, Kure, Hiroshima, 7370023 Japan; 2 Department of Orthopaedic Surgery, Graduate School of Biomedical Sciences, Hiroshima University: 1-2-3, Kasumi, Minami-ku, Hiroshima, 7348551 Japan; 3 Department of Artificial Joints and Biomaterials, Graduate School of Biomedical Sciences, Hiroshima University, Hiroshima, Japan

**Keywords:** p38 mitogen-activated protein kinase, human telomerase reverse transcriptase, malignant fibrous histiocytoma, liposarcoma

## Abstract

**Background:**

One of the major components of telomerase is the human telomerase reverse transcriptase (hTERT) as the catalytic protein. hTERT mRNA expression are reported to be associated with prognosis and tumor progression in several sarcomas. However, there is no clear understanding of the mechanisms of hTERT in human sarcomas. Recent studies have suggested that signals transmitted through p38 mitogen-activated protein kinase (MAPK) can increase or decrease hTERT transcription in human cells. The purpose of this study was to analyse the correlation between p38 MAPK and hTERT in sarcoma samples.

**Methods:**

We investigated 36 soft tissue malignant fibrous histiocytomas (MFH), 24 liposarcomas (LS) and 9 bone MFH samples for hTERT and p38 MAPK expression. Quantitative detection of hTERT and p38 MAPK was performed by RT-PCR.

**Results:**

There was a significant positive correlation between the values of hTERT and p38 MAPK in all samples (r = 0.445, p = 0.0001), soft tissue MFH (r = 0.352, p = 0.0352), LS (r = 0.704, p = 0.0001) and bone MFH samples (r = 0.802, p = 0.0093). Patients who had a higher than average expression of p38 MAPK had a significantly worse prognosis than other patients (p = 0.0036).

**Conclusions:**

p38 MAPK may play a role in up-regulation of hTERT, and therefore, p38 MAPK may be a useful marker in the assessment of hTERT and patients' prognosis in sarcomas.

## Background

Telomerase, an enzyme related to cellular immortality, stabilizes telomere length by adding DNA repeats onto telomere ends [1,2]. Many studies have revealed that telomerase activity is expressed in many different types of carcinomas, detected in more than 85% of the human carcinoma samples, and it has been found to be useful as a prognostic indicator [3-5]. Telomerase activity is mainly regulated by human telomerase reverse transcriptase (hTERT), which is the catalytic subunit of telomerase [6,7]. Also, hTERT has been significantly detected in many types of sarcoma samples, and previous reports have indicated that hTERT expression is associated with tumor aggressiveness, feature and clinical outcome in sarcomas [8-14]. Therefore, hTERT may play an important role in telomere maintenance mechanisms in human sarcomas. However, it is notable that thus far, there has been no clear understanding of the mechanisms of hTERT expression especially in sarcomas. p38 is a mitogen-activated protein kinase (MAPK) activated by phosphorylation on serine/threonine residue when cells are exposed to cellular stress, and has a wide variety of biological functions [15-17]. Recent studies have suggested that signals transmitted through MAP kinase can increase or decrease hTERT transcription in response to various stimuli, depending on the downstream mediators [18-22]. This study was undertaken to analyze the clinical significance of p38 MAPK and hTERT expression in primary tumor samples from soft tissue malignant fibrous histiocytomas (MFH), liposarcomas (LS) and bone MFH patients. In addition, with the broader aim of discovering regulation factors of hTERT in sarcomas, we investigated whether there is a correlation between hTERT and p38 MAPK.

## Methods

### Patients and tumor samples

A total of 69 (36 soft tissue MFHs, 24 LSs and 9 bone MFHs) sarcoma samples were obtained at the time of surgery, were immediately frozen and stored at -80°C until commencement of our study. Summarized clinical data at the time of last observation are shown in Tables 1, 2 and 3. All patients with these sarcomas were treated with tumor resection and/or chemotherapy between 1988 and 2005. We performed brachytherapy or external radiation therapy following conservative surgery for all soft tissue sarcoma patients who received marginal resection. Chemotherapy comprised of multiagent systemic chemotherapy in metastatic patients. High dose ifosfamide, doxorubicin and/or cisplatin were used. We collected all primary tumor samples by tumor resection or biopsy, and no patients had undergone chemotherapy before surgical specimens were collected. The study was approved by our institutional review board (Dai eki 133, and 263).

**Table 1:** Data in 36 patients with soft tissue MFH

Age (Yrs)	Gender	Site	Histol. Type	Prognosis	Period (mos.)	hTERT	p38
53	Male	thigh	stori-pleo	DOD	12	28.4	0
48	Male	thigh	myxoid	NED	80	1564.5	0
76	Female	thigh	stori-pleo	DOD	22	2365	8.7
54	Male	thigh	stori-pleo	DOD	12	978.4	6.1
49	Male	upper arm	stori-pleo	DOD	18	22	2.8
63	Female	axillary	myxoid	CDF	28	383.4	4.5
82	Male	thigh	stori-pleo	CDF	80	181.9	3.3
66	Female	thigh	stori-pleo	CDF	60	133.2	0
75	Male	thigh	stori-pleo	NED	35	1986.5	2.8
45	Female	inguinal	myxoid	CDF	27	8.5	0.3
78	Female	thigh	stori-pleo	DOD	9	8.9	5.2
35	Male	thigh	stori-pleo	CDF	52	1.9	2.1
81	Male	thigh	stori-pleo	CDF	26	0	0
84	Male	buttock	stori-pleo	CDF	26	45.9	10
57	Female	shoulder	stori-pleo	CDF	62	158.3	36.2
76	Female	thigh	stori-pleo	DOD	6	196.8	50.1
75	Male	thigh	stori-pleo	DOD	10	147.3	15.6
57	Male	thigh	stori-pleo	CDF	94	696.5	14.1
69	Male	thigh	stori-pleo	CDF	94	18	60.3
72	Male	thigh	stori-pleo	DOD	49	0	0.3
64	Female	buttock	myxoid	DOD	10	2.6	10.3
55	Female	thigh	myxoid	DOD	21	1029.5	23
59	Female	shoulder	stori-pleo	DOD	47	2656	71.1
74	Male	thigh	myxoid	DOD	27	15.6	0.4
59	Female	lower leg	inflammatory	CDF	115	4.6	1.7
46	Male	thigh	stori-pleo	CDF	98	0	0
73	Male	thigh	stori-pleo	CDF	112	0	0
62	Female	forearm	myxoid	CDF	138	145.3	5
59	Female	thigh	stori-pleo	DOD	7	45.3	1.3
49	Male	upper arm	stori-pleo	CDF	87	10.1	0
85	Male	thigh	stori-pleo	CDF	106	0.9	0.2
58	Female	buttock	stori-pleo	DOD	6	103.8	0.1
73	Male	thigh	stori-pleo	CDF	112	145.3	0
78	Male	lower leg	stori-pleo	CDF	119	125.1	0.2
71	Female	lower leg	myxoid	NED	65	31.9	2.4
73	Female	lower leg	myxoid	CDF	25	135.6	7.8

stori-pleo = storiform-pleomorphic type

CDF = continuously disease-free

NED = no evidence of disease

DOD = died of disease

**Table 2:** Data in 24 patients with liposarcoma

Age (Yrs)	Gender	Site	Histol. Type	Prognosis	Period (mos.)	hTERT	p38
65	Male	thigh	myxoid	NED	93	4	0.4
35	Female	popliteal	myxoid	CDF	108	31.6	1
50	Female	thigh	myxoid	CDF	102	0	0.4
42	Male	shoulder	myxoid	CDF	41	726.6	30.1
65	Male	thigh	myxoid	CDF	56	484.9	38.2
66	Female	thigh	dediff.	CDF	66	271.8	0.2
47	Female	thigh	myxoid	CDF	84	117.5	21.1
58	Male	thigh	myxoid	CDF	76	331.9	0.5
74	Male	thigh	myxoid	DOD	27	148.7	11.2
60	Male	thigh	pleomorphic	CDF	132	145	0.4
51	Male	thigh	pleomorphic	CDF	31	3.1	1.4
66	Male	upper arm	myxoid	CDF	70	29.5	0.7
69	Male	thigh	myxoid	DOD	13	331.2	14
41	Male	lower leg	myxoid	CDF	51	0.8	1.8
47	Male	forearm	dediff.	DOD	12	435.8	2
62	Female	thigh	myxoid	CDF	62	76.5	0.6
68	Male	thigh	myxoid	CDF	100	97.5	1.1
73	Female	buttock	myxoid	DOD	14	391.8	31.6
48	Female	forearm	myxoid	CDF	132	0	1.9
52	Female	thigh	myxoid	CDF	85	91.3	0
48	Male	thigh	myxoid	DOD	15	94.3	0.7
60	Female	thigh	myxoid	CDF	85	58.7	2
36	Male	thigh	myxoid	CDF	81	46.8	0.9
56	Male	thigh	myxoid	CDF	69	191.6	1.2

defiff. = dedifferentiated CDF = continuously disease-free

DOD = died of disease

**Table 3:** Data in 9 patients with bone MFH

Age (Yrs)	Gender	Site	Histol. Type	Prognosis	Period (mos.)	hTERT	p38
23	Female	femur	stori-pleo	CDF	130	304	0
65	Female	femur	stori-pleo	DOD	37	1405.4	191.1
46	Male	femur	stori-pleo	CDF	141	921.8	36.2
27	Female	clavicle	stori-pleo	CDF	92	323.1	10.3
57	Male	femur	stori-pleo	CDF	93	241.7	0
69	Male	femur	stori-pleo	DOD	8	1278.2	60.3
67	Male	sacrum	stori-pleo	DOD	7	324.5	35.2
38	Male	humerus	stori-pleo	DOD	18	603.6	49.3
57	Female	ilium	stori-pleo	DOD	6	326.5	35

stori-pleo = storiform-pleomorphic type

CDF = continuously disease-free

DOD = died of disease

### Quantification of hTERTand p38 MAPK mRNA expression

Total cellular RNA was extracted using a Rneasy Mini Kit (Qiagen, Valencia, CA), and cDNA was synthesized using 1 μg of total RNA using a Transcriptor First Strand cDNA Synthesis Kit (Roche Applied Science, Mannheim, Germany). Quantitative detection of hTERT mRNA and p38 MAPK was performed with the LightCycler TaqMan Master using the LightCycler instrument (Roche Molecular System, Alameda, CA). The primer pairs 5'-CGGAAGAGTGTCTGGAGCAA-3' and 5'-GGATGAAGCGGAGTCTGGA-3' for hTERT, and 5'-ATGCCGAAGATGAACTTTGC-3' and 5'-TCTTATCTGAGTCCAATACAAGCATC-3' for p38 MAPK were used for amplification. PCR used 10 seconds at 95°C, 30 seconds at 60°C and 1 second at 72°C with 45 cycles. Expression of the gene glyceraldehyde-3-phosphate dehydrogenase (GAPDH) was also analyzed in each tumor sample as an indicator of RNA quality. A 3 × 10^6 ^of HeLa cell was used as a positive control. Quantification of mRNA expression was indicated by measuring mRNA expression levels of hTERT or p38 MAPK/mRNA levels of the Hela cell ratio.

### Statistical analysis

The cumulative prospective of overall survival was calculated using the method of Kaplan-Meier. Statistical significance of the differences between the survival curves was evaluated using the log-rank test. Pearson's product-moment correlation coefficient (r and p values) was used to study the relationship between p38 MAPK and hTERT. Data are presented as the mean ± SD. In all analyses, a p value of < 0.05 was considered to indicate significance.

## Results

### Overall results of 69 samples

#### p38 MAPK and hTERT mRNA expression

p38 MAPK expression was demonstrated in 84.1% (58 of 69) and hTERT mRNA expression was demonstrated in 91.3% (63 of 69) of all 69 samples. The levels of p38 MAPK were 13.4 ± 27.7 (range: 0-191.1) and those of hTERT were 336.5 ± 554.8 (range: 0-2656.0) in all samples. We previously reported the data of hTERT in bone and soft tissue MFHs [23,24].

#### Correlation between levels of p38 MAPK and hTERT mRNA expression

**Figure 1:**
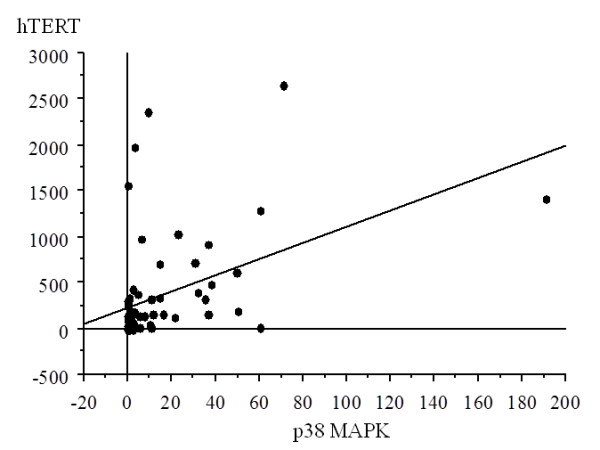
**Correlation between p38 and hTERT in all samples**. There was a significant correlation between the values of p38 expression and those of hTERT, with increased p38 expression with higher hTERT in all samples (r = 0.445, p = 0.0001).

There was a significant correlation between the values of p38 MAPK expression and hTERT, with increased p38 MAPK expression with higher hTERT in all samples (r = 0.445, p = 0.0001) (Figure 1).

#### Prognostic factors

**Figure 2:**
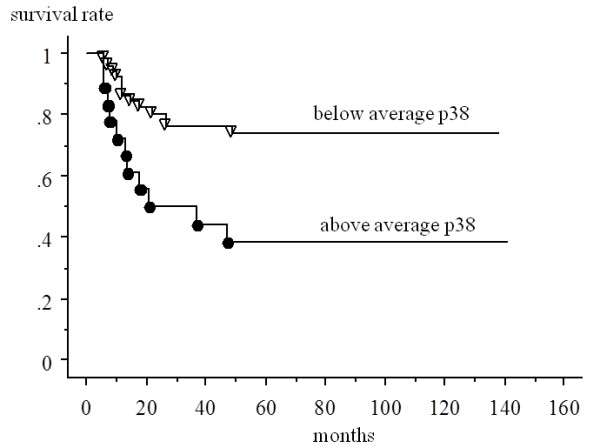
**Kaplan-Meier analysis of the association between the survival and the p38 in all samples**. Patients who had a higher than average expression of p38 MAPK had a significantly worse prognosis (5-year survival rate; 38.1%) than other patients (73.8%) overall (p = 0.0036).

Patients who had a higher than average expression of p38 MAPK had a significantly worse prognosis (5-year survival rate; 38.1%) than other patients overall (73.8%) (p = 0.0036) (Figure 2). There were no significant differences in prognosis between patients who had a higher than average expression of hTERT (5-year survival rate: 38.6%) and those who did not (71.1%) (p = 0.0585).

### Soft tissue MFH samples

#### p38 MAPK and hTERT mRNA expression

p38 MAPK expression was demonstrated in 77.8% (28 of 36) and hTERT mRNA expression was demonstrated in 88.9% (32 of 36) of soft tissue MFH samples. The levels of p38 MAPK were 9.60 ± 17.5 (range: 0-71.1) and those of hTERT were 371.6 ± 695.9 (range: 0-2656.0).

#### Correlation between levels of p38 MAPK and hTERT mRNA expression

**Figure 3:**
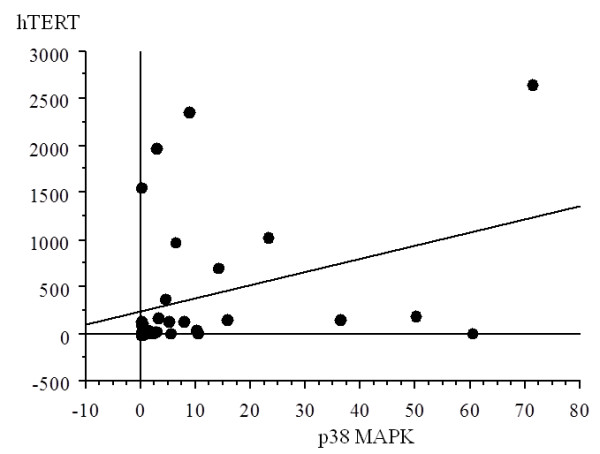
**Correlation between p38 and hTERT in soft tissue MFH samples**. There was a significant correlation between the values of p38 expression and those of hTERT (r = 0.352, p = 0.0352).

There was a significant correlation between the values of p38 MAPK expression and hTERT, with increased p38 MAPK expression with higher hTERT in soft tissue MFH samples (r = 0.352, p = 0.0352) (Figure 3).

#### Prognostic factors

There were no significant differences in prognosis between patients who had a higher than average expression of p38 MAPK (5-year survival rate: 41.7%) and those who did not (65.0%) (p = 0.213). There were no significant differences in prognosis between patients who had a higher than average expression of hTERT (41.7%) and those who did not (62.7%) (p = 0.610).

### Liposarcoma samples

#### p38 MAPK and hTERT mRNA expression

p38 MAPK expression was demonstrated in 95.8% (23 of 24) and hTERT mRNA expression was demonstrated in 91.7% (22 of 24) of LS samples. The levels of p38 MAPK were 6.81 ± 11.5 (range: 0-38.2) and those of hTERT were 171.3 ± 189.9 (range: 0-726.6) in LS samples.

#### Correlation between levels of p38 MAPK and hTERT mRNA expression

**Figure 4:**
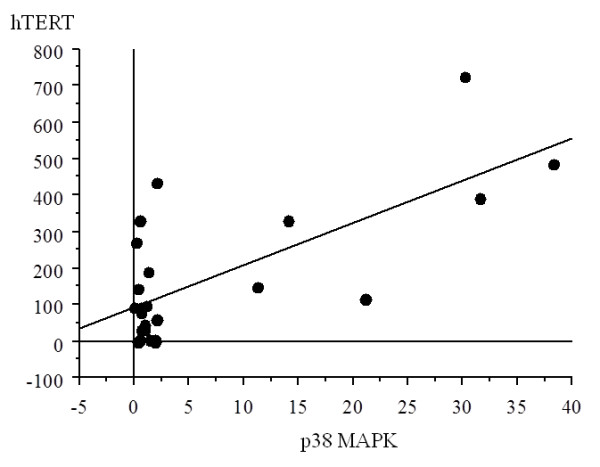
**Correlation between p38 and hTERT in liposarcoma samples**. There was a significant correlation between the values of p38 expression and those of hTERT (r = 0.704, p = 0.0001).

There was a significant correlation between the values of p38 MAPK expression and hTERT, with increased p38 MAPK expression with higher hTERT in LS samples (r = 0.704, p = 0.0001) (Figure 4).

#### Prognostic factors

Patients who had a higher than average expression of p38 MAPK (5-year survival rate: 50.0%) had a significantly worse prognosis than other patients (88.9%) (p = 0.0448) in LS patients. There were no significant differences in prognosis between patients who had a higher than average expression of hTERT (62.5%) and those who did not (87.5%) (p = 0.110).

### Bone MFH samples

#### p38 MAPK and hTERT mRNA expression

p38 MAPK expression was demonstrated in 77.8% (7 of 9) and hTERT expression was demonstrated in all (9 of 9) of bone MFH samples. The levels of p38 MAPK were 46.4 ± 58.2 (range: 0-191) and the levels of hTERT were 636.5 ± 453.3 (range: 241.7-1405.4) in bone MFH samples.

#### Correlation between levels of p38 MAPK and hTERT mRNA expression

**Figure 5:**
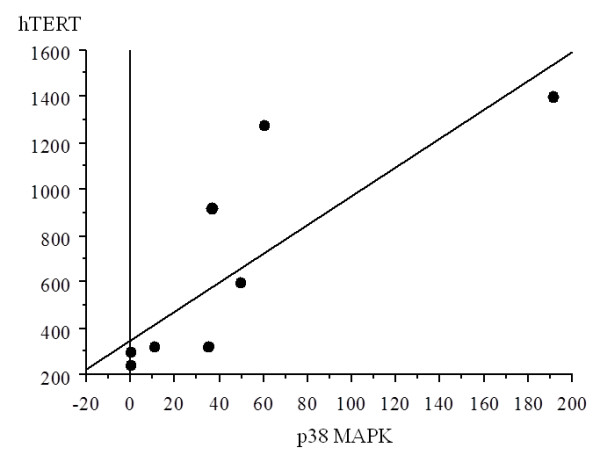
**Correlation between p38 and hTERT in bone MFH samples**. There was a significant correlation between the values of p38 expression and those of hTERT (r = 0.802, p = 0.0093).

There was a significant correlation between the values of p38 MAPK expression and hTERT, with increased p38 MAPK expression with higher hTERT (r = 0.802, p = 0.0093) (Figure 5).

#### Prognostic factors

Patients who had a higher than average expression of p38 MAPK (5-year survival rate: 0%) had a worse prognosis than other patients (66.7%), but did not reach significant differences (p = 0.202). There were no significant differences in prognosis between patients who had a higher than average expression of hTERT (33.3%) and those who did not (50.0%) (p = 0.904).

## Discussion

hTERT is the catalytic telomerase subunit component that copies a template region of its functional RNA subunit to the end of the telomere. In terms of carcinomas, hTERT mRNA expression and telomerase activity are closely associated, and quantification of hTERT mRNA has been reported as an alternative to the measure of telomerase activity [7,25,26]. Also, in sarcomas, the correlation between telomerase activity and hTERT has been reported [9,10,27]. However, in contrast, previous reports maintained that hTERT expression does not correlate to telomerase activity [12,23], and hTERT mRNA expression was only studied in the absence of detectable telomerase activity on sarcomas [8,12,27,28]. There is no clear understanding of the discordance between hTERT and telomerase activity in sarcomas [23,29]. Recently, the presence of telomerase activity and alternative lengthening of telomeres (ALT) in several sarcomas was examined extensively, and these studies indicate a positive correlation between the telomere maintenance mechanism and tumor aggressiveness in several sarcoma types [29]. Furthermore, a positive correlation between hTERT and tumor aggressiveness in several sarcomas has been reported [8-14]. Therefore, it could be necessary to analyze hTERT, in order to elucidate the telomere maintenance mechanisms and the tumorigenesis of sarcomas.

The predominence of large numbers of protein kinases involved in signal cascades following genotoxic stress is the p38 MAPK [30]. p38 MAPK is shown to induce a wide variety of intracellular responses, with roles in tumorigenesis, cell-cycle regulation, development, inflammation and apoptosis [15-17]. Recent studies have suggested that signals transmitted through MAP kinase can regulate hTERT transcription. Epidermal growth factor (EGF) affects the up-regulation of hTERT transcription through the MAP kinase cascades [20]. E26 transformation-specific (Ets) transcription factors, downstream of the mitogen signaling pathways of MAP kinase, regulates hTERT [31]. p38 MAPK may play an important role in the activation of the hTERT promoter by the upstream stimulatory factor (USF) in tumor cells [32]. In the present study, there was a significant positive correlation between the values of p38 MAPK expression and hTERT, with increased p38 MAPK expression with higher hTERT in sarcoma samples. This is the first report to show a correlation between the levels of hTERT mRNA expression and the levels of p38 MAPK in human sarcomas, and these results may suggest that p38 MAPK plays a role in up-regulation of hTERT in soft tissue MFH, liposarcomas, and bone MFH, while we do not have a clear understanding if some factor regulates both p38 MAPK and hTERT expression.

Recent studies have demonstrated that p38 MAPK has diverse roles in the pathogenesis of several cancers and have shown that they are also involved in regulating other functions including the differentiation and proliferation of various cell types [33]. The p38 MAPK pathway is most frequently associated with a tumor suppressor function, based on its negative regulation of proliferation and survival of cells [33,34]. However, contradictory effects have been observed, a fact that points to the pathway playing a positive role in cell-cycle progression in some carcinoma cells [35-37]. In terms of sarcoma cells, inhibition of p38 MAPK activity rescues the antitumor agent fenretinide-mediated cell death in Ewing's sarcoma family of tumors [38], and inhibition of p38 signals results showing a significant reduction in chondrosarcoma cell proliferation mediated by complex effects of p38 signaling on cell-cycle gene expression [39], which suggests that p38 MAPK may play an important role in tumorigenesis in these sarcomas. In the clinical setting, p38 MAPK expression correlates to poor prognosis (p = 0.0036) in overall patients; of high expression of p38 MAPK, indicating the likelihood of a poor outcome and may indicate a positive role of p38 MAPK in tumor proliferation and aggressiveness, in patients with sarcomas. In terms of bone and soft tissue MFH, there were no significant differences in prognosis between patients who had a higher than average expression of p38 MAPK and those who did not. However, patients who had above average p38 (5-year survival rate: soft tissue MFH; 41.7%, bone MFH; 0%) had a worse prognosis than other patients (5-year survival rate: soft tissue MFH; 65.0%, bone MFH; 66.7%), but did not reach significant differences. These results may be due to small numbers of patients. Patients who had a higher than average expression of p38 MAPK (5-year survival rate: 50.0%) had a significantly worse prognosis than other patients (88.9%) (p = 0.0448) in LS patients. Therefore, high expression of p38 MAPK may correlate with a worse prognosis especially for LS patients.

## Conclusions

p38 MAPK may be a useful marker in the assessment of hTERT and prognosis. Given that more than 80% of sarcomas express p38 MAPK and hTERT, elucidation of the pathways and target genes of p38 MAPK in sarcomas will yield additional understandings into the pathogenesis of several sarcomas and may lead to novel therapeutic strategies for their treatment.

## Competing interests

The authors declare that they have no competing interests.

## Authors' contributions

TM, SS, TK, JF, TS carried out literature research, experimental studies and data acquisition, participated in the study design, and drafted the manuscript. YY and OM proposed the study, participated in the design and coordination and helped to draft, and assisted writing the manuscript. All authors read and approved the final manuscript.
